# Ear, Nose, and Throat Foreign Bodies in the Paediatric Population: Did the COVID-19 Lockdown Change Anything?

**DOI:** 10.7759/cureus.27892

**Published:** 2022-08-11

**Authors:** Joshua Garg, Francis De Castro, Paramesh Puttasidiah

**Affiliations:** 1 Otolaryngology, Morriston Hospital, Swansea, GBR

**Keywords:** lock-down, foreign body in throat, foreign bodies in ear or nose, pediatric, covid 19

## Abstract

Background

In the pediatric population, the ear, nose, and throat (ENT) foreign body is a common presentation for emergency departments (ED) and ENT units. COVID-19 has led to a significant impact on the health care system and the overall mental well-being of the general population. With the health care system under significant strain, we noted a continued presence of children with foreign bodies, with some requiring removal under a general anesthetic.

Aim

We aimed to assess if lockdown measures increased or decreased the incidence of children presenting to the hospital with ear, nose, and throat foreign bodies and to evaluate their management by the ED and ENT specialties.

Method

A retrospective data of children presenting with a foreign body in the ear, nose, and throat from March 2020 to August 2020 was compared with the data for the same period in 2019.

Results

Our study showed an overall decrease in children presenting with foreign bodies in 2020 compared to 2019 (n=90 and n=106, respectively). However, the number of children needing general anesthetic remained the same, and those presenting with foreign bodies in the upper aerodigestive tract were higher in 2020.

Conclusion

Children with foreign ear, nose, and throat bodies continued to present to the hospital during the COVID-19 lockdown. Our study shows an overall decrease in the number of children presenting with Ear, Nose, and Throat foreign body during the lockdown, but not statistically significantly different.

## Introduction

Children often present to accident and emergency (A&E) or their general practitioner with a foreign body inserted into the ear, nose, or ingested/inhaled into the aerodigestive system. Most foreign bodies found in the ear and nose are removed in A&E by the emergency physicians [1-3], and the rest are referred to the ENT department. It is estimated that foreign bodies account for approximately 11% of cases in Ear, nose, and throat (ENT) emergency services [4-6]. However, some children need a general anesthetic for extraction. A general anesthetic is usually necessary when the child is uncooperative, if the foreign body is impacted in the ear canal or nasal cavity, or if an ingested foreign body is lodged within the aerodigestive tract. Foreign bodies, such as batteries and inhaled objects, must be removed in an emergency as they can be life-threatening [7].

Pediatric foreign body removals under general anesthetic (GA) involve organizing and a multi-disciplinary team approach. The impact of COVID-19 has resulted in emergency theatres having to run at a reduced capacity, dealing with life-threatening emergencies and urgent cancer cases [8]. Additional measures also have to be put in place concerning aerosol-generating procedures (AGPs), often meaning the increased time between cases due to the potential risks of respiratory virus transmission [9-11]. Children requiring general anesthetic to remove a foreign body add a burden to an already strained service.

This project aims to analyze the incidence of ENT-related foreign bodies in children in a single health board. A six-month "pre-pandemic" period (March-August 2019) will be compared with the same timeframe in 2020 (during the pandemic) to see if the COVID-19 lockdown has had any effect on the frequency of presentations to hospitals. In the United Kingdom, the first national lockdown from March 2020 prohibited all non-essential travel and limited people to one period of exercise per day. It also involved the complete closure of schools/daycare centers, with all non-essential work to be done at home where possible. Potentially, this altered supervision of children could have impacted the number of ENT foreign bodies. We hypothesized that due to potential "fears" of going to hospital due to the pandemic and increased parental supervision at home, the number of attendances would be lower in 2020 compared to 2019 and that potentially, parents/carers would persevere more in attempts to remove foreign bodies.

## Materials and methods

Retrospective data collection was performed to identify all the pediatric cases (16 years old or less) presenting with a foreign body in the ear, nose, or throat. Cases were retrieved from the beginning of March 2020 (the start of the first COVID-19 lockdown) to the end of August 2020. The same data was collected and compared to the beginning of March 2019 and the end of August 2019 (pre-lockdown). The patients were collected from the IT department's coding system with the criteria set to the mentioned timeframes, aged 16 and under, and foreign bodies. This data was initially screened to filter out ENT cases.

To ensure patients weren't missed, departmental records (i.e., inpatient lists) were analyzed. Additionally, the admissions diaries for the pediatric assessment units were analyzed to ensure that all potential patients were identified. The number, anatomical position, and management outcomes (including who managed the patient or if they required theatre) were analyzed. This was done using the online clinical portal and, in some cases, the physical clinical notes/emergency department paperwork. This ensured the coding was correct and the outcomes were accurate for each patient. 

Following this, with all data assumed to be correct, there was a slight chance of patients being missed and that there would be a representative sample size for both timeframes. Statistical analysis included the Chi-squared test in calculating relevant p-values and ascertaining the statistical significance of the data (i.e., p-value <0.05). 

## Results

**Figure 1:**
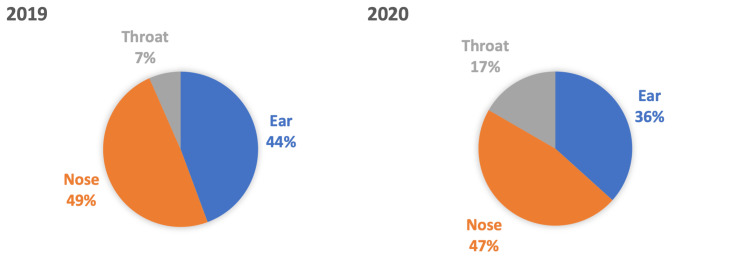
Anatomical location of foreign bodies. There was a similar frequency in the anatomical location of reported foreign bodies. There was over double the incidence in the throat in 2020 compared to 2019 (n = 15 and 7, respectively); however, there was no statistically significant difference (p = 0.076).

One hundred six children presented with a foreign body in the ear, nose, or throat between March-August 2019. For the same period in 2020, there were 90 children, and there was no statistically significant difference (p-value = 0.25). In 2019, 71 (66.98%) patients were five years old or less; in 2020, these figures were 61 (67.77%). Figure 1 shows each study period's anatomical locations (i.e., ear, nose, or throat). There was no statistically significant difference in anatomical locations between 2019 and 2020, including in the throat, where there were 7 cases in 2019 and 15 cases in 2020 (p-value = 0.076).

**Figure 2:**
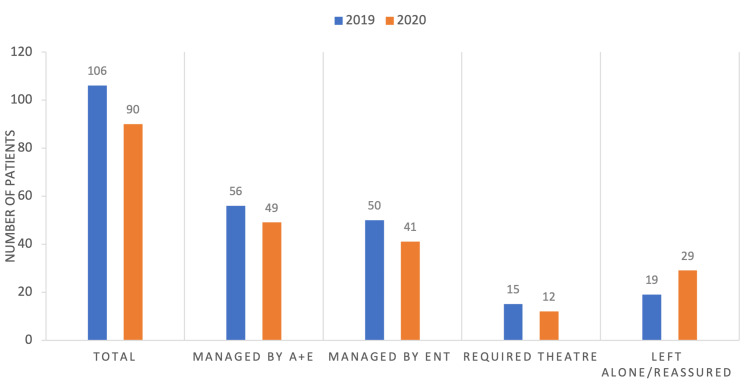
Outcomes/management of foreign bodies. There was no statistically significant difference in how foreign bodies were managed between the 2019 and 2020 patient groups (i.e., whether they were managed by ED vs. ENT or needed theatre).

Figure 2 shows the management/outcomes of the ENT foreign bodies in the two study periods (please see a graph for numbers). There was no statistically significant difference between those being managed by ED versus ENT (p = 0.821) or those requiring a general anesthetic (p = 0.868) between the 2019 and 2020 study periods. Fifteen children required a general anesthetic to remove a foreign body in 2019 and 12 in 2020. There was no statistically significant difference between the years for numbers being managed by A&E vs. ENT (p = 0.821) or those requiring general anesthetic/theatre (p = 0.868). In 2019, 19 patients (17.92%) were examined and reassured that no foreign body was visualized. Of the 19 patients, five were referred to ENT, resulting in the same outcome. For 2020, 29 patients (32.22%) were reassured by A&E, with 15 referred to ENT. 

**Figure 3:**
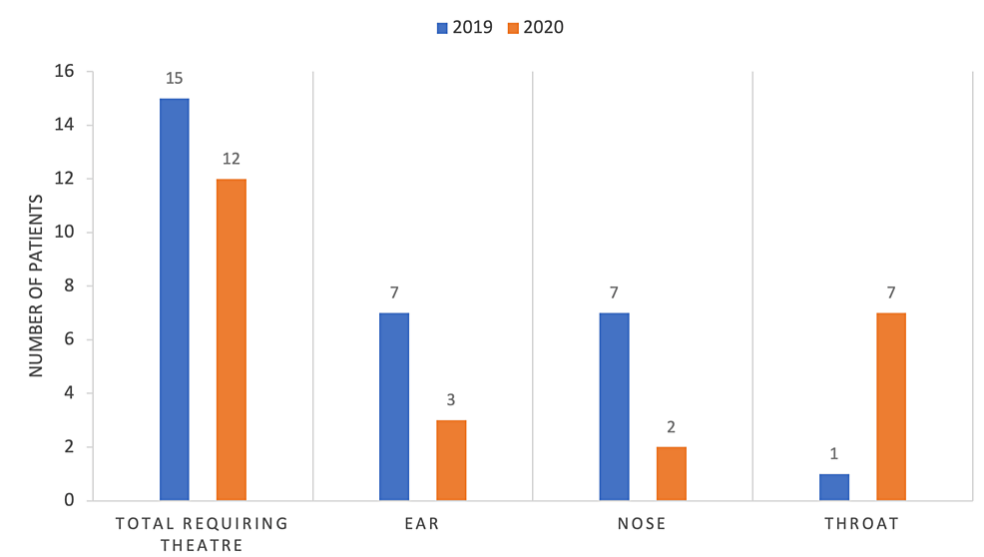
Anatomical locations of foreign bodies in patients requiring theatre.

Some nasal foreign body cases were resolved with the "mother's/parents kiss" in A&E. The "mother's kiss" is a method used to remove nasal foreign bodies whereby a parent will expel air in a particular way into the patient's airways [12]. In 2019, six patients (11.53%) had the foreign body removed via this method. In 2020, there were nine patients (21.43%) where this was successful. Figure 3 shows a breakdown of the theatre cases and their respective anatomical locations. 

## Discussion

Children are commonly present to the general practitioners and A&E department with a foreign body in the ear, nose, or throat. Over 4000 foreign bodies are removed annually from nasal and aural cavities in the UK [13]. 

We hypothesized that due to potential "fears" of going to hospital due to the pandemic, the number of attendances would be lower in 2020 compared to 2019 and that potentially, parents/carers would persevere more in attempts to remove foreign bodies. However, there was no statistical significance in our study.

Our study showed that most foreign bodies were in the nose and the ear, with the nasal foreign bodies being higher in both the 2019 and 2020 cohorts, similar to that seen in a previous study [14]. The proportion of patients with foreign bodies in the throat/airway was higher during the lockdown period in 2020 (16.7%) compared to 2019 (6.6%); however, there was no statistical significance. 

In some cases of nasal foreign bodies, the "mother kiss" proved to be an effective method of removal in 11.53% and 21.43% of cases in 2019 and 2020, respectively. A prospective study reported a 64.5% success in the removal of nasal foreign body with the 'Parent's kiss' technique [12].

In our study, 53.6% of the foreign bodies in both cohorts were managed successively by A&E, and the rest referred to ENT. A study conducted in Australia showed that the majority of the foreign bodies in the ear, nose, and throat were successfully removed in the emergency department in 89% of the cases, and the remaining 11% were referred to ENT specialty, but the study included both children and adults [1]

Children who had an impacted foreign body in the ear or nose with failed retrieval attempts, and some of those with a foreign body in the upper aerodigestive tract, required a general anesthetic for their removal. In our study, 14.1% underwent a general anesthetic in 2019 and 13.3% in 2020 for foreign body removal. In 2020, more patients (n=7) with a foreign body in the throat required emergency surgery than in 2019 (n=1). Although not statistically significant, more ear and nose cases went to a theatre in 2019; however, these were often managed on elective/planned theatre lists as they ran at normal capacity without the pandemic. Suspected tracheobronchial foreign bodies should be investigated/removed on an emergency basis due to the increased risk of mortality [15].

Additionally, button batteries in the ear, nose, or throat can cause increased morbidity due to the potentially corrosive nature of their contents [15]. There was a case of one child who had ingested a button battery in our study. This was removed in the theatre, and the child was subsequently intubated and transferred to the pediatric intensive care unit (PICU), where they fully recovered. 

The strengths of our study included a comprehensive review of the ENT foreign body cases in our center, with a direct comparison between pre-lockdown and intra-lockdown numbers. The main limitation was a limited sample size, which could be improved by conducting a multi-center study or comparisons with other years.

## Conclusions

Our study shows no significant change in the number of children presenting with a foreign body in the ear, nose, or throat during the peak of the COVID-19 lockdown in our center. We hypothesized that potentially the incidence would decrease due to higher levels of supervision or the "fear" of going to the hospital during a pandemic. However, children (with their parents) continue to present to the hospital with foreign bodies in significant numbers despite the COVID-19 pandemic. Additionally, the number of patients requiring theatre was unchanged, resulting in an already overstretched theatre capacity adding an extra burden. Despite the statistical analysis, it shows that foreign bodies are still a significant strain on ENT and emergency departments. Further education for children and parents could improve this. Overall, this study suggests that COVID-19 appears to have had no impact on the incidence of pediatric foreign bodies in ENT presenting to hospital. Multi-center studies and comparisons between more years would provide more significant sample numbers and potentially more insight into this joint hospital presentation. 
